# The Impact of COVID-19 Pandemic on Workplace Accidents in Korea

**DOI:** 10.3390/ijerph18168407

**Published:** 2021-08-09

**Authors:** Eun-Mi Baek, Woo-Yung Kim, Yoon-Jeong Kwon

**Affiliations:** 1 Department of Preventive Medicine, College of Medicine, Catholic University of Korea, Seoul 06591, Korea; hanel2004@naver.com; 2 Department of Economics, Kongju National University Korea, Gongju 32588, Korea; 3 Department of Public Health, College of Medicine, Catholic University of Korea, Seoul 06591, Korea; ssokbak@naver.com

**Keywords:** COVID-19, workplace accidents, gender, industry

## Abstract

(1) Background: Although the number of people infected with COVID-19 has increased over time, its effects on workplace accidents are still poorly understood. On the one hand, COVID-19 can reduce workplace accidents through contracted economic activities or changes in work methods. On the other hand, it can increase workplace accidents by spreading in the workplace. The main purpose of this paper is to examine how COVID-19 affected workplace accidents in Korea during the early part of the pandemic. (2) Methods: This paper utilizes the administrative data on workplace accidents in Korea collected by Korea Occupational Safety and Health Agency. In particular, we use monthly data from February 2016 to August 2020. This period was chosen to rule out the effect from the Middle East Respiratory Syndrome outbreak in 2015 and to include COVID-19 effects in 2020, given the available data. To examine the impact of COVID-19 on workplace injury and illness, we estimate fixed effects regression models, allowing us to control for group and time effects. (3) Results: COVID-19 was generally found to reduce workplace accidents in Korea, particularly through a reduction in occupational diseases. However, we also found that COVID-19 increased occupational injuries for males and workers in the transportation industry. We provide some evidence that these workers experienced an increase in workload and were unable to change work methods including working from home during the COVID-19 pandemic. (4) Conclusions: Our results indicate that to reduce workplace accidents, government interventions should be directed at workers who are unable to change work methods and who are likely to suffer an increase in work burden due to COVID-19.

## 1. Introduction

After the World Health Organization (WHO) declared COVID-19 as a global pandemic on 11 March 2020, the number of confirmed cases grew to approximately 153 million in more than 200 countries by 30 April 2021 [1]. Following the first confirmed cases of COVID-19 in Korea on 20 January 2020, the daily number of infected people was stable compared to other countries. This low level of confirmed cases demonstrated that Korea was one of the most successful countries in containing the spread of COVID-19. However, since August 2020, the number of confirmed cases has increased over the winter and the pattern of repeating declines and spreads continues with the cumulative number of confirmed cases exceeding 100,000 in April 2021 [2].

The Korean government responded to the COVID-19 infection in a systematic and timely manner, which included emphasizing social distancing policy and issuing guidelines to contain infectious diseases in public places and workplaces. As infections in the workplace spread in industries, such as call centers and warehouses, Korean society felt disturbed for a while. Nevertheless, after learning from the Middle East Respiratory Syndrome (MERS) outbreak in 2015, the Korea Disease Control and Prevention Agency (KDCA) responded by providing and distributing essential policies and detailed social distancing measures through daily broadcasting. Further, it developed Business Continuity Planning (BCP), a flexible working plan, early detection of workers’ diseases, and industry-specific guidelines for quarantine management in the workplace. This kind of management, conducted through various activities, has also been proposed by the KDCA and the Korea Occupational Safety and Health Agency (KOSHA) [3]. Due to this effective quarantine management, Korea was able to contain the spread of COVID-19 in the early stage and to maintain a lower number of COVID-19 cases compared to other countries.

In terms of workplace accidents in Korea during the COVID-19 pandemic, the death rate measured by the number of deaths per 10,000 workers increased by 0.01 between December 2019 and December 2020. On the other hand, the workplace accident (injury and disease) rate decreased by 0.01% during the same period. Among these accidents, the number of injured persons decreased by 1664 (1.8%) while the number of sick persons increased by 801 (5.3%) in the same period.

The changes in workplace accidents during the COVID-19 pandemic exhibited different patterns across countries. For example, workplace accidents in the UK did not increase significantly and the number of self-reported occupational injuries decreased in 2020. However, the number of work-related diseases in the UK rose from 200,000 to 1.6 million compared to 2019, and the cases of work-related stress, depression and anxiety were reported to increase from 200,000 to 800,000, while the number of musculoskeletal disease cases remained the same as in 2019 [4].

In Japan, the number of deaths from workplace accidents decreased by 5.1% in 2020 while the number of occupational injuries increased by 4.4% compared to the previous year. By industry, workplace accidents in manufacturing and construction industries decreased, but those in the land freight transportation and service industries increased during the COVID-19 pandemic [5,6]. In Singapore, the number of workplace injuries decreased by 2429 cases from 2019 to 2020, which is a reversal of the increasing trend observed in previous years. However, occupational diseases were reported to have increased by 11 cases in 2020 [7]. In Hong Kong, the number of occupational injuries and diseases in 2020 decreased from 2019 [8]. This is different from the fact that in 2019, occupational injuries and diseases had not changed significantly from the previous year [9]. Lastly, in Taiwan, the decrease in general injuries in 2020 was also known to contribute most to the decrease in workplace injuries [10].

It is evident that there is no consistent pattern observed regarding how COVID-19 has affected workplace accidents across countries. However, there are several routes by which COVID-19 tends to influence workplace accidents. The first, and probably most important factor, is the reduction in economic activities during the COVID-19 period. Due to social distancing and lockdown, the level of production decreased in 2020 [1] and it could have resulted in a reduction of the number of occupational injuries and diseases.

Secondly, changes in work methods may also have affected workplace accidents. Due to the pandemic, many people were forced to reduce working hours and work from home [11,12]. These changes in work methods resulted in less time spent in the workplace and less interaction with other workers and customers, which is likely to reduce injuries and diseases in the workplace. However, workers in the health care and welfare sectors became more vulnerable to occupational injuries and diseases. Note that an increase in part-time work and working from home does not necessarily imply that workplace accidents will decrease because the workload for people who continued working may increase. Employees working in call centers or warehouses are some examples of this.

**Figure 1:**
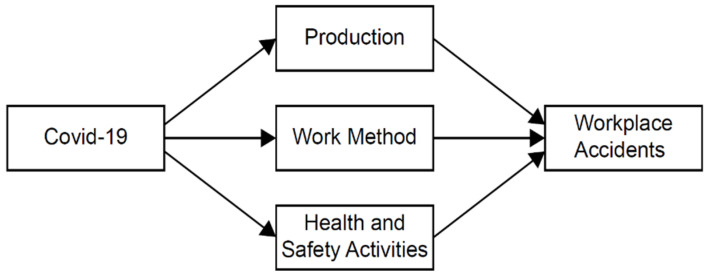
Pathways between COVID-19 and workplace accidents.

Finally, despite the quarantine management supported by government agencies such as KDCA and KOSHA, the financial resources of many companies, especially small ones, became strained from the reduction in production and profits during the COVID-19 period, so the resources and manpower devoted to health and safety activities within such companies might have diminished. This may have caused a rise in workplace accidents. Figure 1 depicts several routes by which COVID-19 could affect these accidents.

The purpose of this study is to examine how COVID-19 affected workplace accidents in Korea. One of the most important features of this paper is that we were able to use monthly panel data which allowed for detailed examination of changes in workplace accidents over a short period of time. The methodology used by Lemieux et al. [13] is extended to a regression analysis to estimate the impact of COVID-19 on occupational injuries and diseases. In estimation, we control for changes in production activities so that we can eliminate the impact of COVID-19 on workplace accidents resulting from a contraction in production.

## 2. Materials and Methods

### 2.1. The Occupational Injury and Disease Data

To estimate the effects of COVID-19 on workplace accidents, we use monthly statistics of occupational injuries and diseases reported from 2016 to 2020. This data set is obtained from KOSHA, and we also use employment data published by the National Statistical Office of Korea to discuss our obtained results.

The official data on occupational injuries and diseases in Korea have been annually collected since 1975. The observations used in this study are accidents (injuries and diseases) that occurred in the workplace requiring medical care for more than four days and that were approved to be related to work according to the Industrial Accident Compensation Insurance. We exclude deaths caused by occupational injuries and diseases because they can be the result of workplace accidents which occurred a long time ago. For example, the death of a mining worker from lung cancer can result from many years working in poor working conditions. Publicly open data are only available on an annual basis. However, since COVID-19 has been around for only a short period of time, annual data are not appropriate to examine the effects of COVID-19 on workplace accidents. Therefore, we utilize the administrative data on workplace accidents authorized by KOSHA. In particular, we use monthly data from February 2016 to August 2020. This period is chosen to avoid the effect of the MERS outbreak which took place in 2015 and to include those of COVID-19 in 2020, given the permitted data.

Our data set provides information on the number of individuals who reported as injured or sick in the workplace as well as their personal and workplace characteristics, such as gender, age, firm size, and industry. We use this information to detect the group-specific effects of COVID-19 on workplace accidents.

### 2.2. A Regression Model

Our main statistical model to be estimated is a fixed effects regression model, which is a regression version of Lemieux et al. [13]. In other words, controlling for personal and workplace-invariant unobserved heterogeneity, we compare the growth rates of workplace accidents (occupational injuries and diseases) in the period of COVID-19 and those in the period without COVID-19 by estimating a fixed effects model.

Specifically, we divide individuals who were reported as injured or sick in the workplace by gender (2 groups), age (5 groups), industry (4 groups) and firm size (6 groups), so we construct 240 (= 2 × 5 × 4 × 6) possible cells per month. The five age groups are comprised of age 15–29, age 30–39, age 40–49, age 50–59, and age >60. The four industries are comprised of mining and manufacturing, construction, transportation and telecommunication, and other services. Lastly, the six firm sizes are comprised of firms with less than 10 employees, 10–29 employees, 30–49 employees, 50–99 employees, 100–299 employees, and >300 employees. We count the number of accidents (injuries and diseases) in each cell; thus, the maximum number of observations is 240 per month because some of the cells are empty, i.e., there is no injury or illness reported in some cells. Therefore, we have unbalanced panel data.

Specifically, our model is specified as

(1) $$\;\ln Y_{ijkn,t,m}-\ln Y_{ijkn,t-1,m}=\alpha +\beta Covid19_{t,m}+\gamma \left(lnecon_{t,m}-lnecon_{t-1,m}\right)+v_{ijkn}+\tau_{m}+\tau_{y}+\varepsilon_{ijkn,t,m}$$

where $Y_{ijkn,t,m}$ is the number of workplace accidents in a group of gender *i,* age group *j*, industry *k*, firm size *n* in year *t* and month *m*. The year (*t*) is from 2016–2020 and month (*m*) is from February to August since the COVID-19 started affecting the Korean economy seriously in February 2020 [2].$\;Covid19_{t,m}$ is a dummy variable which equals 1 if *t* = 2020 and *m* = February…August, 0 otherwise. $econ_{t,m}$ is an aggregate production index which reflects economic conditions at *t* and *m.* This index shows the level of production activities and is provided by Korean Statistical Information Service [3]. Finally, $v_{ijkn}$ is unobserved group-specific heterogeneity captured by a fixed effect term, $\tau_{m}$ and $\tau_{y}$ indicate month and year effects, and $\varepsilon_{ijkn,t,m}$ is an error term.

Model (1) is estimated using fixed effects estimation technique with standard errors corrected for possible heteroscedasticity [4]. Note that the dependent variable is the log difference of workplace accidents, so the coefficient of $Covid19_{t,m}$ (β) indicates the change in the growth rate of workplace accidents over one year, and γ measures the elasticity of the growth rate of workplace accidents with respect to the growth rate of aggregate production index.

As the COVID-19 effect on workplace accidents can vary depending on personal and workplace characteristics, we also estimate Equation (1) for all sub-groups separately. For example, we estimate (1) for males and females, separately. In this case, gender subscript *i* is dropped from (1) so that we control for $v_{jkn}$ as well as $\tau_{m}$ and $\tau_{y}$. The same procedure is applied when we estimate (1) by age group, industry, and firm size, respectively.

## 3. Results

### 3.1. The Summary Statistics of COVID-19 Effects on Workplace Accidents

The number of workplace accidents (injuries and diseases) in Korea for period of 2016–2020 is presented in Table 1. We count workplace accidents from February to August each year since the cases of COVID-19 substantially increased in February 2020. Therefore, we want to compare workplace accidents in 2020 with those in previous years. August is chosen because data are available up to August 2020. Figures for males, females, industry, and firm size are the number of workplace accidents.

For both sexes, the number of workplace accidents decreased by 1.58% between 2019 and 2020. The number of occupational diseases increased by 7.84% while the number of injuries decreased by 3.03%. The decrease in workplace accidents is more conspicuous for female than for male workers. However, we cannot attribute these changes to the effect of the COVID-19 pandemic alone. Consider, for example, the changes in occupational diseases for both sexes. The number of workers with occupational diseases increased by 7.84% between 2019 and 2020. However, it increased even more by 41.14% between 2018 and 2019 [5]. In fact, the rate of change in occupational diseases is the lowest for 2019–2020 during the past 4 years.

To estimate the effect of COVID-19 on workplace accidents, we adopt a DID (Difference in Difference) method used by Lemieux et al. [13]. That is, we calculate

(2) $$\mathrm{COVID}19\;\mathrm{Effect}=(Y_{2020}-Y_{2019})/Y_{2019}-(Y_{2019}-Y_{2018})/Y_{2018},$$

where $Y_{t}$ is the number of workplace accidents in year t from February to August. Equation (2) indicates that the COVID-19 effect is obtained by the growth rate of workplace accidents between 2019 and 2020, subtracted by the growth rate between 2018 and 2019 (the growth rate of normal situation). These effects are presented in the second last column of Table 1. We also calculate the COVID-19 effect by subtracting the average growth rate of 2016–2019 to avoid abnormalities involved in 2018–2019. The figures are provided in the last column of Table 1. Although the magnitudes of COVID-19 and COVID-19(a) are different, the directions of the effects are not so different from each other. Therefore, we use the COVID-19 effect for the following explanations.

The last two columns of Table 1 show that COVID-19 may have contributed to reducing workplace accidents in Korea, especially for female workers with occupational diseases. The reduction in workplace accidents is the most succinct for younger workers, which means that older workers have become more vulnerable to COVID-19. COVID-19 also reduced workplace accidents in most industries except for the agricultural and transportation industries. For the agricultural industry, the number of workplace accidents is relatedly small, and increased only by 14 from 939 in 2019 to 953 in 2020. Hence, the COVID-19 effect is maybe overstated. Conversely, in the transportation industry, the number of workplace accidents increased by 715 from 2019 to 2020. We also decomposed accidents into injuries and diseases for this industry and found that injuries increased by 682 while diseases increased by 33. We believe that the increase in injuries in transportation is due to an increased workload for workers in this industry during the COVID-19 pandemic. Finally, COVID-19 is shown to reduce workplace accidents more for large companies although the pattern is not so clear.

The last row of Table 1 shows the index of production activities for each year [6]. The index increased from 2016 to 2018 but decreased sharply by 1.41% between 2019 and 2020. The COVID-19 effect on production activities is estimated to be −1.29%. Obviously, the decrease in production activities is associated with the decrease in workplace accidents because a contraction in production is likely to reduce employment and working hours, which in turn reduces the number of injured and sick people in the workplace.

### 3.2. Estimates of Regression Model

#### 3.2.1. Total Workplace Accidents

Workplace accidents consist of occupational injuries and diseases. Thus, we estimate model (1) for total number of accidents, injuries, and diseases separately and the results are presented in Table 2. Columns (2), (4) and (6) include the growth rate of aggregate production index to control for the change in economic activities. The bottom of Table 2 presents the number of groups included in estimation. For injuries, 226 (gender × age × industry × firm size) groups are used, while for diseases, 174 groups are included, which means that more injuries than diseases were reported in our constructed groups from 2016–2020. Group-fixed effects are included in all estimations.

The coefficients of the COVID-19 dummy are all negative and statistically significant at the 1% level. COVID-19 decreased the growth rate of total accidents by about 10%, and its impact was particularly large for occupational disease, decreasing it by about 21%. This result is consistent with the COVID-19 effects presented in Table 1 and is similar to the case of Hong Kong [8].

Interesting results are observed when we add the growth rate of aggregate production index in the model. Columns (2), (4) and (6) show that the coefficients of the COVID-19 dummy become smaller and their statistical significances decrease considerably. For example, the COVID-19 dummy for total injuries now becomes statistically insignificant even at the 10% level. These results indicate that the negative impact of COVID-19 on workplace accidents is to a large extent due to a decrease in economic activities. However, for occupational disease, the COVID-19 dummy is still statistically significant at the 5% level. This implies that besides the decrease in economic activities, the COVID-19 pandemic may have changed the way people work such as “work from home” so that it reduced occupational diseases. Social distancing may also have played a role in reducing illness at workplaces due to reduced contact among workers.

The COVID-19 effects on workplace accidents presented in Table 2 are acquired by comparing the growth rates of workplace accidents in 2020 with those in 2019 since the omitted year is 2019 in estimation. Another way to measure the COVID-19 effect is to compare the growth rate of workplace accidents in 2020 with those in 2016–2019, which may be considered as an average effect presented in Table 1. We estimate the same model without including year dummies, and the results are presented in Table 3.

Although the magnitude and statistical significance of the COVID-19 dummies decrease in Table 3 compared to those in Table 2, the qualitative conclusion remains the same. That is, the COVID-19 pandemic decreased the growth rate of total accidents, especially the growth rate of occupational diseases. Again, adding the growth rate of aggregate production index in the model considerably reduces the magnitudes of the COVID-19 effects, which reemphasizes the importance of economic activities in explaining changes in workplace accidents. In addition, it is the occupational disease that was most influenced by COVID-19, even when we controlled for the growth rate of aggregate production index.

#### 3.2.2. Workplace Accidents for Various Groups

COVID-19 can have unequal impacts on workplace accidents depending on individual characteristics as well as types of industries and firms, therefore, we estimate model (1) by gender, age group, industry and firm size separately. Table 4 presents the estimation results for males and females, respectively.

The results are very intriguing. For males, COVID-19 increased the growth rate of injuries while for females, the opposite is true. As we discussed in the introduction, COVID-19 can affect workplace accidents through changes in work method or workload, or changes in financial and manpower devoted to health and safety measures, in addition to changes in production level. These changes may be brought into the workplace because of social distancing and companies’ lack of financial resources.

Some recent studies showed that COVID-19 affected male and female workers differently [14,15]. Although the employment effect of COVID-19 differs between male and female workers depending on studies conducted, the general consensus is that female workers with children became more family-oriented and burdened by more family responsibilities due to school closure [15,16]. Conversely, the role of male workers in the workplace becomes more important, and a reduction in employment due to COVID-19 may have increased the work burden for male workers, leading to an increase in injuries. Table 4 shows that the adverse effect of COVID-19 on injuries is more serious for male than for female workers, which implies that male workers might have received an excessive workload compared to female workers. However, the effect of COVID-19 on occupational disease is similar for both males and females, although the effect is not statistically significant for females.

The estimation results for various age groups are presented in Table 5. The negative effect of COVID-19 on the growth rate of workplace accidents seems to be larger as workers get older. This is different from the COVID-19 effects presented in Table 1, which are obtained without controlling for changes in economic activities and other group-fixed effects. For the youngest group, COVID-19 decreased the growth rate of accidents by 4.6% while for the oldest group, COVID-19 decreased by 8.2%. Therefore, the results in Table 5 indicate that the workload for younger workers may have increased while working hours decreased more for older workers. Further, “work from home” may have been implemented more extensively for older workers during the COVID-19 pandemic. In fact, labor statistics in Korea show that between 2019 and 2020, the weekly working hours for workers aged >50 decreased by 5.8% while those for aged 15–29 decreased by 4.9%.

As for injuries, the coefficients of the COVID-19 dummy are all negative; they are small and statistically insignificant. However, for occupational diseases they become larger and statistically significant at least at the 10% level for the age 30–39, 40–49 and >60 groups. These estimates again confirm that the impact of COVID-19 is greater for occupational diseases than for injuries, and it is also concentrated on prime and older age groups rather than young workers.

Next, we estimate model (1) for the four industries and provide the results in Table 6. We use manufacturing, construction and service production indices when estimating respective sectors rather than using the aggregate production index for all industries. There is no information on the production index for transportation and telecommunication industry, thus the service production index is used for this sector.

We observe very different patterns of the COVID-19 impact on workplace accidents among industries. For manufacturing and construction industries, COVID-19 decreased the growth rate of workplace accidents, not only through occupational disease but also through injury. On the other hand, for service and transportation industries, COVID-19 increased the growth rate of workplace accidents, especially through injuries.

A relative increase in injuries in transportation and other service industries indicates that the workload due to COVID-19 may have been increased more in those industries. In particular, the employment in delivery and telemarketing industries, which were booming sectors during the COVID-19 period, increased in Korea [7]. In addition, workers in delivery companies in Korea are known to have a high rate of injuries, and thus it has become an important health and safety policy issue in Korea [17]. The results in Table 6 reemphasize the vulnerability of workers in the service sector during the COVID-19 period.

Finally, the estimation results for companies with various employment sizes are presented in Table 7. Although workplace accidents are known to be concentrated in small companies in Korea [18], the growth rate of accidents is not biased toward small companies during the COVID-19 period. Significant negative coefficients of the COVID-19 are only observed on occupational disease for firms with 20–29 and >300 employees. The overall impact of the COVID-19 on workplace accidents is not generally significant for each firm size, nor is any consistent pattern observed by firm size.

## 4. Discussion

This study examines the changes in workplace accidents in Korea over the past several years to identify the impact of COVID-19. We used the DID method proposed by Lemieux et al. [13] to provide an average effect of COVID-19 on workplace accidents and estimate a fixed-effects model, in which we controlled for changes in production activities as well as group-specific effects. We found that COVID-19 generally reduced occupational injuries and diseases in Korea. However, the effects of COVID-19 were found to vary depending on individual and firm characteristics, especially by gender and industry. In Korea during the COVID-19 pandemic, the growth rate of injuries of male workers increased while the opposite was true for females. We suspect that COVID-19 may have increased the work burden of male workers because female workers were burdened with more family responsibilities following school closures [15,16]. We also found that the growth rate of workplace accidents in Korea, especially occupational injuries, in the service sectors including the transportation industry increased during the COVID-19 period.

The effects of COVID-19 estimated in this study were obtained by controlling for changes in production activities. Therefore, the difference in the impact of COVID-19 between male and female workers probably arose from differences in work burdens, work methods, and health and safety resources including union protection or health and safety committee coverage in the workplace. The union density of male workers in Korean establishments is higher than that of female workers like in many other countries, and male workers in Korea are more likely to be covered by exclusive health and safety committees [19]. Therefore, it is unlikely that injuries of male workers increased more than those of female workers during the COVID-19 period because male workers received less protection from a union or health and safety committee than female workers.

In terms of work burden, labor statistics in Korea show that females lost more jobs than males and worked fewer hours per week than males during the COVID-19 period. Table 8 presents the number of workers and weekly working hours for males and females in Korea for 2016–2020.

Between 2019 and 2020, the number of female workers decreased by 1.1% while the number of male workers decreased by 0.5%. For the same period, female weekly hours decreased by 5.6% while male weekly hours decreased by 4.6%. Therefore, it can be fairly stated that the work burden for male workers increased because more male workers had to work and spend longer hours in the workplace during the COVID-19 period, which might increase the chance of injury.

Concerning the changes in work method including “work from home”, there is no official data in Korea to indicate that females, compared to males, changed their working style in a way that prevented workplace accidents from occurring during the COVID-19 period. However, Adams-Prassl et al. [20] estimated the number of jobs that can be done at home in the US and UK and found that females are less likely to work from home [8]. Their results are consistent with the estimates obtained by Lee and Kim [21] who show that female workers are more vulnerable to COVID-19 infection because they are disproportionately more likely to work in health care and welfare sectors, exposing them more frequently to close contact with patients and clients. Therefore, it is unlikely that injuries of females increased less than those of males because females worked from home more extensively. In sum, we conclude that the reason why male workers had a higher growth rate of injuries during the COVID-19 period is because their work burden increased more due to the reduction in employment of, and hours worked by, female workers.

We also examined why the service industry, including the transportation industry, experienced a higher growth rate in injuries than manufacturing and construction industries during the COVID-19 period. As the union density of manufacturing and construction industries is higher than that of the service industry, it is conceivable that a lack of protection from unions is one of the reasons why workers in the service industry had a higher growth in injuries.

Next, to see the changes in workload for workers in transportation and other service industries, we compared the changes in employment and working hours across industries. Table 9 presents number of workers and weekly working hours for manufacturing, construction, transportation, and other service industries in Korea for 2016–2020.

We found that between 2019 and 2020, manufacturing and construction employment decreased by 0.83% and 1.69%, respectively, while transportation employment increased by 1.74%, although employment in other services decreased by 1.68%. We also computed changes in weekly working hours and found that all four industries experienced a similar decrease in working hours of 4–5%. Table 9 suggests that during the COVID-19 period, workers in the transportation industry were more prone to injuries in the workplace than those in manufacturing and construction industries.

Finally, according to Adams-Prassl et al. [20], approximately 44.7% of jobs in the manufacturing industry and 43.2% of jobs in the construction industry in the US and UK can be done at home, while around 37.6% of jobs in the transportation and storage industry can be performed at home. This suggests that workers in the latter industry are more exposed to workplace accidents. In sum, all three factors (union protection, work burden and work method) seem to have worked against the workers in the transportation industry.

Note that the discussion above is based on a heuristic approach. To establish causal relationships between the three factors and an increase in workplace accidents for males and workers in transportation industry during the COVID-19 period, we need a formal regression analysis. That deserves a separate study which we intend to pursue in the future.

## 5. Conclusions

In 2020, the COVID-19 pandemic had a major impact on both the health and economic well-being of people around the world, and its detrimental impact continues today. Although much attention has been paid to how COVID-19 affects the health of people, especially older people, and how to contain the spread of COVID-19 in communities, little research has been done on how COVID-19 has affected occupational health and injuries of people in the workplace. In this paper, we examined the impact of COVID-19 on workplace accidents in Korea.

Using the administrative data on workplace accidents in Korea, collected by Korea Occupational Safety and Health Agency, and estimating fixed effects regression models in which we controlled for group and time effects, we found that COVID-19 generally reduced workplace accidents in Korea, particularly through a reduction in occupational disease. However, we also found that COVID-19 increased occupational injuries for males and workers in the transportation industry. We provided some evidence that male workers experienced an increase in workload while workers in the transportation industry were unable to change work methods including working from home and they also experienced an increase in workload during the COVID-19 period. Our results imply that to reduce workplace accidents, government interventions should be directed and implemented at the individual level to help workers who are unable to change work methods and who are likely to suffer the increased work burden due to COVID-19.

It should be kept in mind that the number of workplace accidents in this paper is not the same as the number of COVID-19 cases that occurred in the workplace. COVID-19 cases were included in workplace accidents as long as they occurred at the workplace and required medical care for more than four days. In this study, we did not intend to count COVID-19 related workplace accidents. Rather, as Figure 1 shows, the COVID-19 pandemic influences workplace accidents through various routes. We measured the overall impact of the COVID-19 pandemic on workplace accidents.

Further, note that the results obtained in this study are short-term effects of COVID-19 as we only looked at the changes in workplace accidents in 2020 from February to August. Since the COVID-19 pandemic is ongoing in Korea as well as in other countries, it is imperative to look at the long-term effects in the future. Nevertheless, our findings suggest that when trying to prevent injuries and diseases in the workplace, the government should collect detailed information regarding ability to change work methods and changes in work burden across occupations during the COVID-19 period.

## Figures and Tables

**Table 1:** Basic statistics of workplace accidents in Korea (February–August 2016–2020).

	2016	2017	2018	2019	2020	2017–2016 (%)	2018–2017 (%)	2019–2018 (%)	2020–2019 (%)	COVID-19	COVID-19 (a)
Both Sexes											
Accidents	51,648	51,135	57,053	61,221	60,255	−0.99	11.57	7.31	−1.58	−8.88	−7.54
Injuries	47,652	46,587	51,268	53,056	51,450	−2.23	10.05	3.49	−3.03	−6.51	−6.79
Diseases	3996	4548	5785	8165	8805	13.81	27.20	41.14	7.84	−33.30	−19.55
Males	41,084	40,074	44,368	46,836	46,674	−2.46	10.72	5.56	−0.35	−5.91	−4.95
Females	10,564	11,061	12,685	14,385	13,581	4.70	14.68	13.40	−5.59	−18.99	−16.52
Age Group											
Age 15–29	4901	5056	5768	6372	6226	3.16	14.08	10.47	−2.29	−12.76	−11.53
Age 30–39	7009	6574	7435	7849	7730	−6.21	13.10	5.57	−1.52	−7.08	−5.67
Age 40–49	10,597	9781	10,674	11,271	10,689	−7.70	9.13	5.59	−5.16	−10.76	−7.50
Age 50–59	16,649	16,091	17,559	18,126	17,245	−3.35	9.12	3.23	−4.86	−8.09	−7.86
Age >60	12,490	13,633	15,617	17,603	18,365	9.15	14.55	12.72	4.33	−8.39	−7.81
Industry											
Agriculture	1290	1039	1005	939	953	−19.46	−3.27	−6.57	1.49	8.06	11.26
Mining	687	759	986	1277	1371	10.48	29.91	29.51	7.36	−22.15	−15.94
Construction	15,148	14,736	15,627	16,650	16,492	−2.72	6.05	6.55	−0.95	−7.50	−4.24
Manufacturing	14,742	14,220	15,147	15,221	14,548	−3.54	6.52	0.49	−4.42	−4.91	−5.58
Transportation	2342	2410	2836	3346	4061	2.90	17.68	17.98	21.37	3.39	8.51
Other service	17,439	17,971	21,452	23,788	22,830	3.05	19.37	10.89	−4.03	−14.92	−15.13
Firm Size											
1–9	25,802	25,495	27,089	28,186	27,339	−1.19	6.25	4.05	−3.01	−7.05	−6.04
10–29	12,193	11,859	13,188	13,832	13,072	−2.74	11.21	4.88	−5.49	−10.38	−9.94
30–49	4471	4216	4618	4881	4657	−5.70	9.54	5.70	−4.59	−10.28	−7.76
50–99	3568	3478	4003	4397	4488	−2.52	15.09	9.84	2.07	−7.77	−5.40
100–299	2884	2978	3916	4628	4962	3.26	31.50	18.18	7.22	−10.96	−10.43
300–499	737	877	1229	1477	1662	19.00	40.14	20.18	12.53	−7.65	−13.91
>500	1993	2232	3010	3820	4075	11.99	34.86	26.91	6.68	−20.23	−17.91
Production index	101.7	105.0	106.5	106.4	104.9	3.26	1.50	−0.12	−1.41	−1.29	−2.96

Note: For both sexes, figures in 2016–2020 are the number of workplace accidents, injuries and diseases that occurred from February to August each year. COVID-19 is calculated as Equation (2) in the text. Other service industries include wholesale and retail trade, health and welfare, food, and accommodation industries.

**Table 2:** Estimation results of fixed effects models.

Variables	Total Accidents		Total Injuries		Total Diseases	
	(1)	(2)	(3)	(4)	(5)	(6)
COVID-19	−0.101 ***	−0.047 *	−0.078 ***	−0.018	−0.212 ***	−0.152 ***
	(0.027)	(0.027)	(0.026)	(0.027)	(0.042)	(0.042)
$\ln econ_{t,m}-lnecon_{t-1,m}$		3.481 *** (0.502)		3.828 *** (0.541)		3.698 *** (0.809)
March	0.265 ***	−0.321 ***	0.261 ***	−0.382 ***	0.194 ***	−0.426 ***
	(0.035)	(0.088)	(0.034)	(0.092)	(0.055)	(0.154)
April	0.101 ***	0.042	0.106 ***	0.042	0.057	−0.006
	(0.032)	(0.031)	(0.032)	(0.031)	(0.049)	(0.050)
May	0.162 ***	−0.029	0.168 ***	−0.042	0.002	−0.194 ***
	(0.033)	(0.041)	(0.033)	(0.043)	(0.046)	(0.067)
June	0.174 ***	−0.094 *	0.180 ***	−0.115 **	0.085 *	−0.196 **
	(0.033)	(0.049)	(0.033)	(0.051)	(0.050)	(0.084)
July	0.259 ***	0.181 ***	0.266 ***	0.180 ***	0.052	−0.037
	(0.030)	(0.030)	(0.031)	(0.031)	(0.049)	(0.054)
August	0.142 ***	0.026	0.148 ***	0.021	−0.031	−0.149 **
	(0.035)	(0.035)	(0.034)	(0.035)	(0.055)	(0.059)
Year 2017	−0.119 ***	−0.225 ***	−0.088 ***	−0.204 ***	−0.222 ***	−0.334 ***
	(0.019)	(0.025)	(0.021)	(0.028)	(0.043)	(0.052)
Year 2018	0.043 *	−0.015	0.066 ***	0.003	−0.101 **	−0.160 ***
	(0.025)	(0.026)	(0.024)	(0.026)	(0.040)	(0.046)
Constant	−0.021	0.158 ***	−0.062 **	0.135 ***	0.276 ***	0.463 ***
	(0.028)	(0.034)	(0.026)	(0.035)	(0.046)	(0.066)
Observations	5366	5366	5287	5287	2976	2976
R-squared	0.038	0.051	0.035	0.049	0.028	0.037
Number of groups	227	227	226	226	174	174

Note: Numbers in parentheses are robust standard errors. Base month and year are February and 2019, respectively. ***, **, and * indicate significance at the 1%, 5%, and 10% level, respectively.

**Table 3:** Estimation results of fixed effects models without year dummies.

Variables	Total Accidents		Total Injuries		Total Diseases	
	(1)	(2)	(3)	(4)	(5)	(6)
COVID-19	−0.082 ***	−0.037	−0.076 ***	−0.015	−0.122 ***	−0.098 **
	(0.022)	(0.025)	(0.022)	(0.025)	(0.031)	(0.039)
$\ln econ_{t,m}-lnecon_{t-1,m}$		1.509 ***		2.057 ***		0.828
		(0.412)		(0.421)		(0.642)
March	0.234 ***	−0.027	0.234 ***	−0.122	0.161 ***	0.019
	(0.034)	(0.075)	(0.034)	(0.078)	(0.056)	(0.125)
April	0.070 **	0.037	0.080 **	0.035	0.024	0.006
	(0.032)	(0.032)	(0.032)	(0.032)	(0.049)	(0.049)
May	0.132 ***	0.042	0.143 ***	0.020	−0.027	−0.074
	(0.033)	(0.040)	(0.033)	(0.041)	(0.046)	(0.061)
June	0.144 ***	0.020	0.154 ***	−0.015	0.054	−0.012
	(0.033)	(0.045)	(0.033)	(0.047)	(0.050)	(0.072)
July	0.228 ***	0.186 ***	0.239 ***	0.183 ***	0.017	−0.007
	(0.030)	(0.031)	(0.031)	(0.032)	(0.049)	(0.052)
August	0.112 ***	0.055	0.123 ***	0.044	−0.064	−0.094 *
	(0.034)	(0.035)	(0.033)	(0.035)	(0.056)	(0.057)
constant	−0.013	0.049 *	−0.041	0.044	0.212 ***	0.246 ***
	(0.026)	(0.029)	(0.026)	(0.030)	(0.038)	(0.044)
observations	5366	5366	5287	5287	2976	2976
R-squared	0.027	0.030	0.025	0.031	0.017	0.017
number of groups	227	227	226	226	174	174

Note: Numbers in parentheses are robust standard errors. Base month is February. ***, **, and * indicate significance at the 1%, 5%, and 10% level, respectively.

**Table 4:** Estimation results of fixed effects models by gender.

	Males			Females		
Variables	Accidents	Injuries	Diseases	Accidents	Injuries	Diseases
COVID-19	0.021	0.057 **	−0.171 ***	−0.145 ***	−0.131 **	−0.112
	(0.026)	(0.027)	(0.049)	(0.054)	(0.052)	(0.084)
$\ln econ_{t,m}-lnecon_{t-1,m}$	3.489 ** (0.440)	3.518 *** (0.484)	2.851 *** (0.956)	3.479 *** (1.121)	4.296 *** (1.165)	5.962 *** (1.429)
Month dummy	yes	yes	yes	yes	yes	yes
Year dummy	yes	yes	yes	yes	yes	yes
Constant	0.097 ***	0.059 *	0.359 ***	0.250 ***	0.251 ***	0.748 ***
	(0.031)	(0.034)	(0.078)	(0.072)	(0.073)	(0.109)
Observations	3212	3206	2157	2154	2081	819
R-squared	0.081	0.074	0.039	0.038	0.042	0.052
Number of groups	120	120	113	107	106	61

Note: Numbers in parentheses are robust standard errors. Base month and year are February and 2019, respectively. ***, **, and * indicate significance at the 1%, 5%, and 10% level, respectively.

**Table 5:** Estimation results of fixed effects models by age group.

	Variables	COVID-19	$\ln econ_{t,m}-lnecon_{t-1,m}$	Constant	Observations	R-Squared	Number of Group
Age 15–29	accidents	−0.046	1.736	0.023	969	0.038	42
	injuries	−0.014	2.183 *	0.031	954	0.035	42
	diseases	−0.193	0.245	0.185	269	0.078	26
Age 30–39	accidents	−0.025	3.844 ***	0.189 ***	994	0.072	42
	injuries	0.013	4.735 ***	0.155 **	977	0.078	41
	diseases	−0.201 *	3.670 *	0.198	486	0.062	36
Age 40–49	accidents	−0.020	3.722 ***	0.079	1062	0.071	47
	injuries	−0.015	4.070 ***	0.087 *	1051	0.065	47
	diseases	−0.186 **	0.765	0.309 **	666	0.043	36
Age 50–59	accidents	−0.055 *	3.341 ***	0.129	1200	0.071	48
	injuries	−0.021	3.255 **	0.064	1189	0.065	48
	diseases	−0.083	6.408 ***	0.769 ***	828	0.060	37
Age >60	accidents	−0.082	4.572 ***	0.350 ***	1141	0.064	48
	injuries	−0.046	4.779 ***	0.323 ***	1116	0.066	48
	diseases	−0.148 *	4.367 ***	0.508 ***	727	0.058	39

Note: Month and year dummies are included in all estimations. ***, **, and * indicate significance at the 1%, 5%, and 10% level, respectively.

**Table 6:** Estimation results of fixed effects models by industry.

	Variables	COVID-19	$\ln econ_{t,m}-lnecon_{t-1,m}$	Constant	Observations	R-Squared	Number of Group
Manufacturing	accidents	−0.101 **	1.078 **	0.058	1560	0.043	60
	injuries	−0.073 **	1.148 **	0.029	1513	0.037	60
	diseases	−0.183 **	1.287 **	0.340 ***	1041	0.063	56
Construction	accidents	−0.156 **	0.522	−0.141	1106	0.076	47
	injuries	−0.145 **	0.532	−0.156	1102	0.075	47
	diseases	−0.446 ***	2.339 **	0.979 ***	479	0.081	29
Transportation	accidents	0.046	1.569	0.175 *	1083	0.030	60
	injuries	0.092	1.231	0.092	1055	0.025	59
	diseases	0.031	3.001	0.105	311	0.080	29
Other Service	accidents	0.060 *	5.477 ***	0.228 ***	1617	0.116	60
	injuries	0.086 **	6.010 ***	0.204 ***	1617	0.117	60
	diseases	−0.030	5.329 ***	0.477 ***	1145	0.039	60

Note: Manufacturing includes mining and transportation includes telecommunication. Other service industries include wholesale and retail trade, health and welfare, food, and accommodation industries. Month and year dummies are included in all estimations. ***, **, and * indicate significance at the 1%, 5%, and 10% level, respectively.

**Table 7:** Estimation results of fixed effects models by firm size.

No. of Workers	Variables	COVID-19	$\ln econ_{t,m}-lnecon_{t-1,m}$	Constant	Observations	R-Squared	Number of Group
1–9	accidents	−0.052	4.265 ***	0.029	955	0.1398	38
	injuries	−0.033	4.564 ***	0.009	954	0.1444	38
	diseases	−0.110	2.683 *	0.316 **	653	0.0285	31
10–29	accidents	−0.040	5.020 ***	0.166 *	919	0.0937	38
	injuries	−0.028	4.779 ***	0.100	914	0.0962	38
	diseases	−0.212 **	5.734 ***	0.626 ***	576	0.0765	28
30–49	accidents	−0.069	1.512	0.031	847	0.0283	38
	injuries	−0.061	1.701	0.035	838	0.0235	37
	diseases	−0.096	1.356	0.152	423	0.0554	28
50–99	accidents	0.007	3.245 ***	0.147 *	856	0.0451	37
	injuries	0.044	3.946 ***	0.106	845	0.0550	37
	diseases	−0.195	4.373 *	0.540 ***	438	0.0526	28
100–299	accidents	0.009	5.329 ***	0.266 **	873	0.0918	38
	injuries	0.030	4.806 **	0.193	863	0.0773	38
	diseases	−0.080	5.299 **	0.692 ***	468	0.0546	28
>300	accidents	−0.126	1.496	0.313 ***	916	0.0607	38
	injuries	−0.055	3.041 **	0.380 ***	873	0.0552	38
	diseases	−0.249 **	1.822	0.417 *	418	0.0638	31

Note: Month and year dummies are included in all estimations. ***, **, and * indicate significance at the 1%, 5%, and 10% level, respectively.

**Table 8:** Number of workers and weekly working hours for males and females in Korea (2016–2020).

	Males				Females			
	No. of Workers (1000)	Change (%)	Weekly Working Hours	Change (%)	No. of Workers (1000)	Change (%)	Weekly Working Hours	Change (%)
2016	15,223		45.0		11,164		39.3	
2017	15,383	1.05	45.0	−0.08	11,354	1.70	39.3	0.22
2018	15,365	−0.12	43.5	−3.18	11,447	0.82	37.9	−3.57
2019	15,455	0.58	42.9	−1.36	11,639	1.68	37.1	−2.27
2020	15,371	−0.54	41.0	−4.60	11,513	−1.09	35.0	−5.64

Note: All statistics are based on February to August each year.

**Table 9:** Number of workers for industries in Korea (2016–2020).

	Manufacturing		Construction		Transportation		Other Service	
	No. of Workers (1000)	Change (%)	No. of Workers (1000)	Change (%)	No. of Workers (1000)	Change (%)	No. of Workers (1000)	Change (%)
2016	4594		1841		3305		11,556	
2017	4568	−0.56	1976	7.35	3278	−0.81	11,749	1.67
2018	4500	−1.49	2012	1.78	3320	1.27	11,816	0.57
2019	4419	−1.80	2016	0.23	3423	3.11	11,918	0.86
2020	4382	−0.83	1982	−1.69	3482	1.74	11,717	−1.68

Note: All statistics are based on February to August each year. Other service industries include wholesale and retail trade, health and welfare, food, and accommodation industries.

## Data Availability

The data used to support the findings of this study were supplied under license and so cannot be made freely available.
